# Anterior quadratus lumborum block for postoperative recovery after total hip arthroplasty: a study protocol for a single-center, double-blind, randomized controlled trial

**DOI:** 10.1186/s13063-020-4090-0

**Published:** 2020-02-05

**Authors:** Masaru Kikuchi, Takahiro Mihara, Yusuke Mizuno, Hiroko Fujimoto, Sachiko Arai, Takeshi Nomura, Takahisa Goto

**Affiliations:** 1 Department of Anesthesiology, Yokohama City University Hospital, 3-9, Fukuura, Kanazawa-ku, Yokohama city, 236-0004 Japan; 2 Department of Pharmacy, Yokohama City University Hospital, 3-9, Fukuura, Kanazawa-ku, Yokohama city, 236-0004 Japan; 3 Department of Intensive Care Medicine, Tokyo Women’s Medical University Hospital, 8-1, Kawada-cho, Shinjuku-ku, Tokyo, 162-0054 Japan

**Keywords:** Anterior quadratus lumborum block, Hip osteoarthritis, Peripheral nerve block, Postoperative pain, Regional anesthesia, Total hip arthroplasty

## Abstract

**Background:**

Appropriate pain management is essential to improve the postoperative recovery after total hip arthroplasty (THA). Various case reports have indicated that anterior quadratus lumborum block (QLB) provides effective postoperative analgesia in lower limb surgeries. However, few randomized controlled trials have confirmed the efficacy of anterior QLB for lower limb surgeries. The aim of this single-center, double-blind, randomized controlled trial is to confirm the efficacy of anterior QLB for postoperative recovery after THA.

**Methods:**

The participants will be randomly assigned to either the anterior QLB or placebo groups, using a set of random numbers for the allocation sequence. Only pharmacists will be aware of the allocations; other investigators will be blinded until study completion. After induction of general anesthesia, anterior QLB will be performed by using 0.25% levobupivacaine or normal saline. Fentanyl will be administered according to blood pressure change during the surgery. The primary outcome will be the quality of recovery 40 score (QoR-40). Secondary outcomes will include the visual analog scale score of pain intensity at rest and movement, intraoperative and postoperative doses of fentanyl, and incidence of postoperative nausea and vomiting. Statistical analysis will be performed by using the Student’s *t* test, Mann–Whitney *U* test, and Fisher’s exact test as appropriate. A *P* value of less than 0.05 will be considered statistically significant.

**Discussion:**

The results of our study will reveal whether anterior QLB is effective for postoperative recovery after THA.

**Trial registration:**

UMIN Clinical Trials Registry, UMIN000032255. Registered on 15 April 2018.

## 1. Background

Hip osteoarthritis is a disease that causes pain and restricts the range of motion as the hip joint deforms. In Japan, the prevalence of hip osteoarthritis is estimated at 1.0%–4.3% and the number of patients with hip osteoarthritis reaches 1.2–5.1 million [1]. The treatment options for patients with hip osteoarthritis include conservative therapies and surgery. Conservative therapies include patient education, hyperthermia treatment, pharmacological treatment using non-steroidal anti-inflammatory drugs, and physical therapy treatment tailored to the patient’s condition. Surgery is recommended for patients whose condition does not improve with conservative therapies or for those with advanced disease [2]. Recently, total hip arthroplasty (THA) has been widely performed; about 23,000 THA surgeries are performed every year in Japan [3].

THA causes moderate to severe postoperative pain, and inadequate perioperative analgesia management delays ambulation and decreases the quality of postoperative recovery [4]. Opioids are commonly used for postoperative analgesia in various surgeries but their use is associated with respiratory depression or postoperative nausea and vomiting (PONV) and impaired quality of recovery. Therefore, regional anesthesia is often preferred.

Regional anesthesia for postoperative analgesia in THA includes epidural anesthesia and peripheral nerve blocks. Although epidural anesthesia has been used for postoperative pain management in THA, it has become restricted as perioperative antithrombotic drugs are generally used for orthopedic patients. Currently, many types of peripheral nerve blocks, such as the femoral nerve block (FNB) and the lumbar plexus block (LPB), are used for THA surgeries. Peripheral nerve block has been thought to cause less serious complications than epidural anesthesia [5]. The efficacy of FNB for THA has been reported in many studies in the past few decades [6, 7]. However, it has been suggested that FNB might not provide sufficient postoperative analgesia for THA because the hip joint is innervated not only by the femoral nerve but also by other nerves such as the obturator nerve and the sciatic nerve. LPB is a procedure during which a local anesthetic is administered around the lumbar nerve roots so that the femoral nerve, obturator nerve, and lateral femoral cutaneous nerve are blocked. Although LPB provides adequate postoperative analgesia for THA [8], it has a high potential of serious complications such as nerve injury and hematoma [9] because the needle tip should be advanced close to the nerves.

The quadratus lumborum block (QLB), first reported by Blanco in 2007, is a compartment block procedure during which a local anesthetic is injected into the muscle plane of the quadratus lumborum muscle under ultrasound guidance [10]. Currently, QLB is divided into four types based on the injection point of the local anesthetic: lateral, anterior, posterior, and intramuscular QLB. QLB is widely used for various types of abdominal surgeries [11]. In the anterior QLB, described by Børglum *et al*. in 2013, a local anesthetic is injected between the quadratus lumborum muscle and the psoas major muscle [12]. Although anterior QLB was first reported for analgesia in abdominal surgeries, it could provide analgesia for lower limb surgeries by spreading the local anesthetic around the psoas major muscle and blocking lumbar nerve roots such as in LPB. Cadaveric studies showed the spread of a dye around the lumbar plexus by anterior QLB [13, 14]. Anterior QLB may block not only the femoral nerve but also the obturator nerve and the lateral femoral cutaneous nerve, thus possibly providing more effective analgesia in THA than FNB. Additionally, because the needle tip is distant from the lumbar nerve roots in anterior QLB, it has a lower risk of nerve injury than LPB. Indeed, reports on serious QLB-related complications are scarce.

The efficacy of anterior QLB for lower limb surgeries has been reported in some case reports [15–17], but no randomized controlled trials (RCTs) have been conducted. Therefore, it remains unclear whether anterior QLB provides sufficient analgesia and improves the quality of postoperative recovery after lower limb surgeries such as THA.

The aim of this single-center, double-blind RCT is to confirm the efficacy of anterior QLB for postoperative recovery after THA. Here, we describe the study protocol for such a trial.

## 2. Methods/Design

### 2.1. Trial design

This study is designed as a single-center, prospective, double-blind RCT. The participants will be randomly assigned to either the anterior QLB group or the placebo group.

### 2.2. Ethics

This study was approved by the institutional review board of Yokohama City University Hospital (B180405008) and was registered with the UMIN Clinical Trials Registry (UMIN000032255, registered on April 15, 2018, https://upload.umin.ac.jp/cgi-open-bin/ctr/ctr_view.cgi?recptno=R000036231). This trial will be performed in accordance with the principles of the Declaration of Helsinki (Edinburgh 2000 version). Written informed consent will be obtained from each participant before enrollment.

### 2.3. Randomization

A pharmacist (SA) will provide a set of 140 random numbers for the allocation sequence using a website (http://www.randomization.com). None of the investigators, except for the pharmacists, will be aware of the block size. The random allocation sequence will be available to pharmacists only and thus it will be concealed from the other research team members. The included participants will be randomly assigned to either the anterior QLB group or the placebo group in a ratio of 1:1.

### 2.4. Blinding

The pharmacists in the Department of Pharmacy of Yokohama City University Hospital will prepare the syringes containing either 30 mL of 0.25% levobupivacaine (Maruishi Pharmaceutical Co., Tokyo, Japan) or 30 mL of normal saline in accordance with the allocation sequence. The syringes will be labeled “trial drug” and given a number and will be transported to the operating rooms by the drug conveyance in the hospital. The pharmacists in the Department of Pharmacy will participate in the trial only at this stage; therefore, the participants, attending anesthesiologist, surgeons, evaluators, and the researchers will be unaware of the random allocation sequence. The random allocation sequence will not be exposed until the final data analysis report is completed.

### 2.5. Study setting

This trial will be conducted in the Yokohama City University Hospital (3–9, Fukuura, Kanazawa-ku, Yokohama city, Japan).

### 2.6. Eligibility criteria

#### 2.6.2. Inclusion criteria

Patients eligible for the study must comply with all of the following criteria at randomization:

- elective unilateral THA;
- age of at least 20 years;
- American Society of Anesthesiologists Physical Status (ASA-PS) 1–2;
- written informed consent to participate in this trial.

#### 2.6.2. Exclusion criteria

- revision cases;
- liver and kidney dysfunction (aspartate aminotransferase of more than 80 IU/L, alanine aminotransferase of more than 80 IU/L, estimated glomerular filtration rate of less than 50 mL/min);
- coagulopathy (prothrombin time/international normalized ratio of more than 1.50, activated partial thromboplastin time of more than 60 s);
- body mass index of more than 35;
- use of a strong opioid, such as morphine or fentanyl;
- allergy to the study drugs and their components;
- dementia;
- cases judged to be inappropriate by the researchers (e.g., cases where the patient has undergone other femoral surgeries such as intramedullary nailing).

Since certain patients with hip osteoarthritis receive tramadol for management of their pain in Japan, we decided to include these patients in our trial and exclude those receiving strong opioids such as morphine or fentanyl.

### 2.7. Interventions

#### 2.7.1. General anesthesia induction and maintenance

When the patient arrives in the operating room, a standard vital monitoring system, which includes electrocardiography equipment, non-invasive blood pressure monitor, and pulse oximeter, will be in place and a 22- or 20-gauge intravenous catheter will be inserted in the patient’s forearm. General anesthesia will be induced with propofol 1.5 mg/kg, fentanyl 3 μg/kg, and rocuronium 0.6 mg/kg; tracheal intubation will be performed. General anesthesia will be maintained using desflurane 3%–5% and remifentanil 0.2 μg/kg per min. The concentration of desflurane and additional use of rocuronium will be left to the discretion of the attending anesthesiologist. If the patient’s blood pressure increases to more than 20% of the baseline blood pressure during surgery, the anesthesiologist will administer fentanyl 50–100 μg and observe the patient for 15 min. On the contrary, if the blood pressure decreases to less than 20% of the baseline blood pressure, the anesthesiologist will administer ephedrine 4–8 mg or phenylephrine 0.05–0.1 mg and observe the patient for 15 min. The attending anesthesiologist will measure the blood pressure every 5 min; in cases with further decline, additional administration of ephedrine or phenylephrine will be permitted.

#### 2.7.2. Anterior QLB procedure

After general anesthesia induction, anterior QLB will be performed in the lateral position. A low-frequency convex probe for the abdomen (2–5 MHz convex probe, Sonosite Edge® rC60xi; Sonosite Canada Inc., Markham, ON, Canada) will be placed horizontally above the iliac crest. On ultrasound, the lumbar vertebral body and transverse process appear as “thumbs up” structures. Additionally, three muscles can be found around the transverse process: the psoas major, erector spinae, and quadratus lumborum muscles. The psoas major is located on the ventral side of the transverse process, the erector spinae is located on the dorsal side of the transverse process, and the quadratus lumborum is located on the lateral side of the transverse process.

**Fig. 1:**
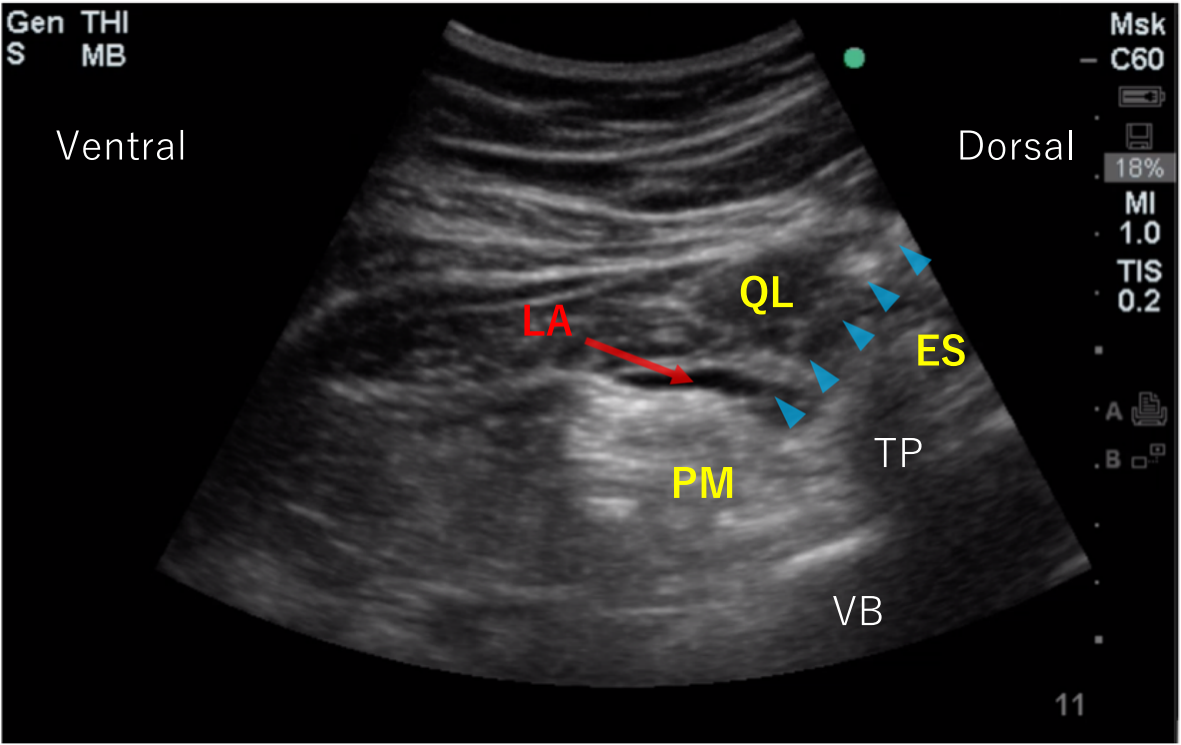
Ultrasound image of anterior quadratus lumborum block (QLB). Triangles indicate needles. *Abbreviations*: *ES* erector spinae muscle, *LA* local anesthetic, *PM* psoas major muscle, *QL* quadratus lumborum muscle, *TP* transversus process, *VB* vertebral body

After a pre-scan and ultrasound parameter optimization, the probe will be applied by using a sterile technique. A 20-gauge short-bevel needle (Visioplex, Vygon, France) will be inserted in the plane from the posterior edge of the convex probe through the quadratus lumborum in an anteromedial direction. When the needle tip reaches the area between the psoas major and the quadratus lumborum, 30 mL of 0.25% levobupivacaine will be injected in the anterior QLB group; the same amount of normal saline will be injected in the placebo group. Figure 1 shows an ultrasound image of anterior QLB.

The procedures of anterior QLB will be performed by the attending anesthesiologists under the guidance of either one of the experts in ultrasound-guided peripheral nerve blocks (MK, YM, HF, and TN). Confirmation by at least two anesthesiologists should ensure that the quality of anterior QLB is maintained.

We will observe whether any complications related to the perioperative procedures, including surgical and anesthetic complications, occur until the end of the trial. Our clinical trial will be performed within the scope of health insurance; therefore, the costs of treatment in participants who experience potential harm will be covered.

#### 2.7.3. Perioperative management of pain and PONV

As multimodal analgesia, 20 mg/kg acetaminophen (maximum of 1000 mg/body) will be administered after the end of surgery and repeated each 6 h until 24 h. From postoperative days 1 to 7, the patients will receive celecoxib 100 mg twice a day. Intravenous fentanyl will be used for patient-controlled analgesia (IV-PCA) using CADD-Solis (Smiths Medical, Grasbrunn, Germany). A bolus injection of 10 μg fentanyl with a 10-min lockout time may be administered for analgesia; continuous infusion of fentanyl will not be considered. In our clinical experience, the pain in most patients may be managed with this protocol. If the patients experience pain, particularly before they start drinking water, flurbiprofen axetil or diclofenac sodium will be administered during the postoperative period. However, in our clinical experience, the incidence of flurbiprofen axetil or diclofenac sodium administration is considerably low.

To prevent PONV, dexamethasone 6.6 mg will be administered before the start of the surgery. Droperidol 0.02 mg/kg or metoclopramide 10 mg may be used according to the postoperative condition. Repeated administration of droperidol and metoclopramide will be permitted in patients continuing to experience PONV.

### 2.8. Outcomes

#### 2.8.1. Primary outcome

The primary outcome will be the Quality of Recovery score 40 (QoR-40) 24 h after surgery. The QoR-40 is a tool that assesses patient-reported outcomes, including quality of life. It was originally developed and validated in Australia in 2000 and is currently used widely as an indicator of postoperative recovery [18, 19]. The QoR-40 is a recovery-specific and patient-rated questionnaire that contains 40 items measuring five dimensions: physical comfort (12 items), emotional state (9 items), physical independence (5 items), psychological support (7 items), and pain (7 items). The total score and subscales of the QoR-40 are measured by using a five-point Likert scale (for positive items: 1 = none of the time, 5 = all of the time; for negative items, the scoring is reversed) and then individual scores are added together; the final score ranges from 40 to 200. In 2011, the Japanese version of the QoR-40 was validated in accordance with standard methods of cultural adaptation and psychometric analysis [20].

#### 2.8.2. Secondary outcomes

The secondary outcomes will be as follows:

- visual analog scale (VAS) score of pain intensity at rest and movement at postoperative days 1 and 2; scores range from 0 (no pain) to 10 (maximal pain) points;
- total dose of fentanyl; the total dose of intraoperative fentanyl and cumulative dose of postoperative fentanyl during the 24 h after surgery are measured separately;
- the time to first use of IV-PCA after surgery;
- intraoperative dose of remifentanil;
- the frequency and dose of other analgesics (i.e., flurbiprofen axetil or diclofenac sodium);
- the incidence of PONV during the 24 h after surgery;
- the time of first ambulation;
- the manual muscle test (MMT) results for the quadriceps muscle during day 1 of postoperative rehabilitation;
- perioperative complications related to surgical or anesthetic procedures (i.e., wound infection, bleeding, dislocation, reoperation, nerve injury, hypotension, or falling);
- days in hospital.

### 2.9. Study timeline and data collection

**Fig. 2:**
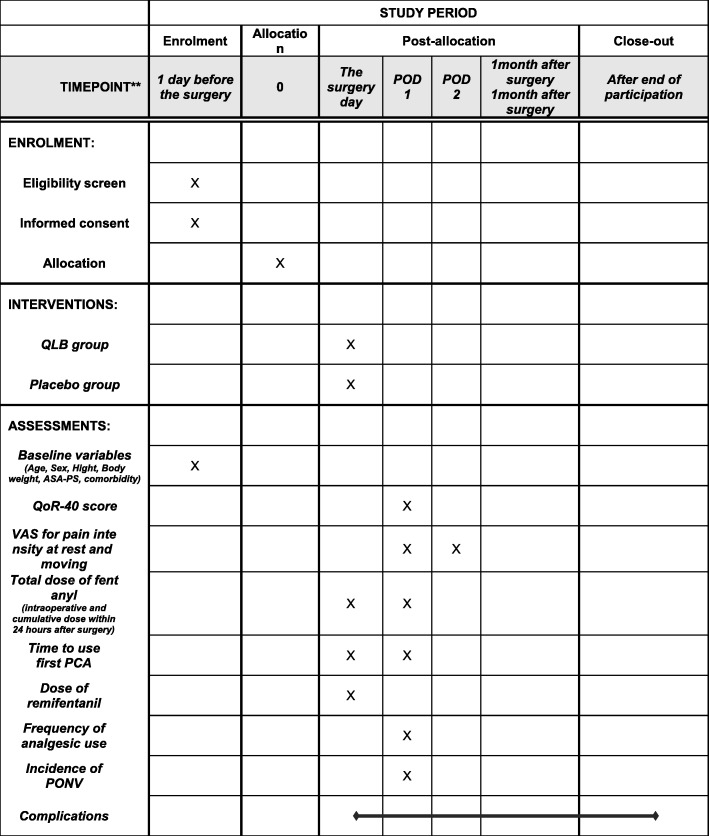
The schedule of enrolment, interventions, assessments, and data collection. *Abbreviations*: *POD* post-operative days, *PONV* postoperative nausea and vomiting, *QLB* quadratus lumborum block, *QoR* quality of recovery

One researcher (MK) will screen the list of operations 1 day before surgery and identify potentially eligible participants. The research coordinator will inform each participant about the aim of the research and the potential risks and benefits. After informed consent is obtained from the participants, their characteristics, including age, sex, height, body weight, body mass index, medical history, comorbidities, ASA-PS classification, and vital signs, will be recorded. We will also record the Apfel score, which includes the following four factors: female gender, no smoking, postoperative use of opioids, and history of PONV or motion sickness. High Apfel scores indicate a high risk of PONV [21]. The timeline for the assessment and data collection regarding primary and secondary outcomes is shown in Fig. 2.

### 2.10. Data protection

Participants in this trial will receive individual registry numbers, and the researchers will manage the collecting data using the numbers. Therefore, private information, including names or IDs, will not be used. The clinical report forms will be kept in a locked safety box, and the researchers will collect and analyze the data by using an off-line personal computer.

### 2.11. Sample size

The sample size was calculated on the basis of the primary outcome, the QoR-40. The minimum clinically important difference of QoR-40 is estimated at 10 points on the basis of previous studies and our clinical experience [18, 22]. The standard deviation (SD) for the QoR-40 was 14 in a previous study [23], and it has been reported that the SD for the QoR-40 in Japan is larger than that in other countries [20]. Therefore, we have estimated an SD for the QoR-40 of 20. A total of 63 patients in each group will be required to detect a 10-point difference, with an alpha error of 0.05 and a beta error of 0.2 (i.e., power of 0.80). Considering dropouts, we plan to enroll 70 patients in each group.

### 2.12. Statistical analysis

Statistical analysis will be performed with R software, a free language and software environment for statistical computing and graphics. All statistical tests will be two-sided, and the level of significance will be set at 0.05. First, continuous data will be analyzed by using the Shapiro–Wilk test to confirm normal distribution. Data with a normal distribution will be expressed as mean ± SD and tested by Student’s *t* test. Data without normal distribution will be expressed as median and interquartile range and tested by the Mann–Whitney *U* test. Categorical data will be tested by using the Fisher’s exact test.

## 3. Discussion

Perioperative pain management is essential for patients undergoing THA [4]. Appropriate pain management improves ambulation time and the quality of postoperative recovery. Among the various regional anesthesia techniques used for perioperative pain management of THA, anterior QLB can be performed in patients taking anticoagulant agents; furthermore, anterior QLB has a low risk of lumbar plexus injury because the needle stays far from the nerve root [12]. Therefore, anterior QLB is thought to be safer than epidural anesthesia and LPB. The efficacy of anterior QLB for lower limb surgeries has been described in case reports [15–17] but has not been confirmed in RCTs. Since anterior QLB was originally described for abdominal surgeries, it is controversial whether it would actually be effective for lower limb surgeries. To provide evidence for the efficacy of anterior QLB for lower limb surgeries, an RCT is necessary. The results of our trial will contribute to provide such evidence. Femoral nerve, fascia iliac compartment, and LPBs are analgesic techniques used during lower limb surgeries. However, about half of the patients in our hospital receive general anesthesia and postoperative intravenous fentanyl without these blocks, as the standard of care for THA, as these techniques are time-consuming and are associated with certain safety concerns. In our double-blind placebo-controlled RCT, all participants will receive postoperative intravenous fentanyl as the standard of care, and we will investigate the efficacy of the anterior QLB. The placebo will be administered to the control group to maintain double-blinding and to reduce bias.

Perioperative pain is only one of the indicators of the quality of postoperative recovery. We believe that improving the quality of postoperative recovery is more important than relieving perioperative pain. Therefore, we selected QoR-40 as the primary outcome in this study.

In this trial, we will perform a single injection, and not continuous injection, during anterior QLB. The reason why we selected the single-injection approach is that continuous injection should be performed only after confirming the benefits of the single injection. This is because continuous nerve blocks are less likely to be effective if the single-injection blocks have no effect.

One of the limitations of our RCT is that we will focus only on short-term outcomes and will not evaluate improvements in long-term outcomes. If our study reveals the short-term efficacy of anterior QLB in THA, further research would be necessary for confirming long-term outcomes.

In conclusion, our single-center, prospective, double-blind RCT will reveal the efficacy of anterior QLB for THA and hopefully provide strong scientific evidence for its use in the postoperative management of THA. Additionally, our results might be extrapolated to other lower limb surgeries and continuous injection blocks (Additional file 1).

### 3.1. Trial status

The trial is currently in the recruitment phase. The first participant was randomly assigned on 21 May 2018. At the time of manuscript submission, we had recruited one third of the calculated sample size. The recruitment will be completed by March 2020. This protocol is version 1.0, dated 13 March 2018. Trial completion is expected by March 2020.

## Supplementary information

**Additional file 1.** SPIRIT (Standard Protocol Items: Recommendations for Interventional Trials) 2013 Checklist: Recommended items to address in a clinical trial protocol and related documents*

## Data Availability

The datasets generated and analyzed during this study are available from the corresponding author on reasonable request.

## Abbreviations

ASA-PS: American Society of Anesthesiologists Physical Status
FNB: Femoral nerve block
IV-PCA: Intravenous patient-controlled analgesia
LPB: Lumbar plexus block
PONV: Postoperative nausea and vomiting
QLB: Quadratus lumborum block
QoR-40: Quality of recovery score 40
RCT: Randomized controlled trial
SD: Standard deviation
THA: Total hip arthroplasty
